# Cervical carotid to vertebral artery high-flow interposition graft bypass serves as an extracranial communicating pathway between anterior and posterior circulation for vertebrobasilar lesions

**DOI:** 10.1007/s00701-025-06709-y

**Published:** 2025-11-27

**Authors:** Xuan Wang, Xiaoguang Tong, Jie Qiao, Minggang Shi, Yanguo Shang, Hu Wang

**Affiliations:** 1 Department of Neurosurgery, Tianjin Huanhu Hospital, Tianjin Medical University, Tianjin, China; 2 Clinical College of Neurology, Neurosurgery and Neurorehabilitation, Tianjin Medical University, Tianjin, China; 3 Department of Neurosurgery, Tianjin Central Hospital for Neurosurgery and Neurology, Tianjin, China; 4 Laboratory of Microneurosurgery, Tianjin Neurosurgical Institute, Tianjin, China; 5 National Stroke College, Tianjin University, Tianjin, China; 6 Tianjin Key Laboratory of Cerebral Revascularization and Head and Neck Neuro-Oncology for Technology Transformation, Tianjin, China; 7 Tianjin Key Laboratory of Cerebral Vascular and Neural Degenerative Diseases, Tianjin, China

**Keywords:** Communicating bypass, Extracranial-extracranial bypass, Extracranial vertebral artery, High-flow bypass, Vertebrobasilar lesion

## Abstract

**Purpose:**

Conventional surgical techniques for posterior circulation bypass have certain limitations, necessitating innovative approaches. This study evaluates the indications and outcomes of cervical carotid-to-V2 vertebral artery bypass—functioning as an extracranial "posterior communicating artery"—in the treatment of vertebrobasilar lesions.

**Methods:**

The V2 bypass procedure via a cervical anterolateral approach was applied in cases of (1) bilateral vertebral artery (VA) occlusions (Type I), (2) subclavian artery (SBA) occlusion with steal phenomenon (Type II), and (3) compensated posterior circulation aneurysm (Type III).

**Results:**

All eight patients exhibited patent bypass grafts. In seven cases with anterior-to-posterior bypass flow, the carotid artery supplied the entire vertebrobasilar territory. All four Type I patients showed improved regional cerebral perfusion. Among the three Type II patients, two (with a non-dominant contralateral VA) underwent VA ligation, resulting in significant reduction of blood steal. In one patient with bilateral symmetric VAs, VA ligation was not performed due to personal reasons, and hemodynamic status remained unchanged. One Type III patient showed contrast retention within the aneurysm sac and progressive shrinkage following bypass.

**Conclusion:**

The V2 bypass uses a short interposition graft between the anterior and posterior circulations, offering high flow and orthograde perfusion without complex skull base manipulation. This technique simplifies revascularization for posterior circulation ischemia, mitigates steal phenomena in SBA occlusion, and enables trans-circulatory flow modulation for compensatory aneurysms.

**Supplementary Information:**

The online version contains supplementary material available at 10.1007/s00701-025-06709-y.

## Introduction

The conventional posterior circulation bypass built tunnels leading to the vertebrobasilar system via cerebellar vessels with recipient transfer, which involves not only deep anastomosis but also an extracranial-intracranial (EC-IC) low-flow bypass with donor arteries from the anterior circulation and mainly focus on their respective focal perforators and distal vascular territories. However, extracranial vertebral artery (VA)-related bypass has further evolved to revolutionize cerebral revascularization [2, 34, 36].

Traditional posterior circulation bypass techniques involve anastomosis to cerebellar vessels with recipient transfer, requiring deep anastomosis and often constituting a low-flow extracranial-intracranial (EC-IC) bypass using anterior circulation donors, focusing on focal perforators and distal territories. Recent advances in extracranial vertebral artery (VA) bypass have revolutionized cerebral revascularization [2, 34, 36].

Conventional posterior circulation bypass procedures establish vascular conduits to the vertebrobasilar system using cerebellar arteries as recipient vessels. This approach typically requires deep anastomosis and functions as a low-flow extracranial-intracranial (EC-IC) bypass, utilizing donor arteries from the anterior circulation that primarily target specific perforating branches and distal vascular territories. In recent years, however, bypass techniques involving the extracranial vertebral artery (VA) have undergone substantial development, leading to transformative advances in cerebral revascularization [2, 34, 36].

**Fig. 1:**
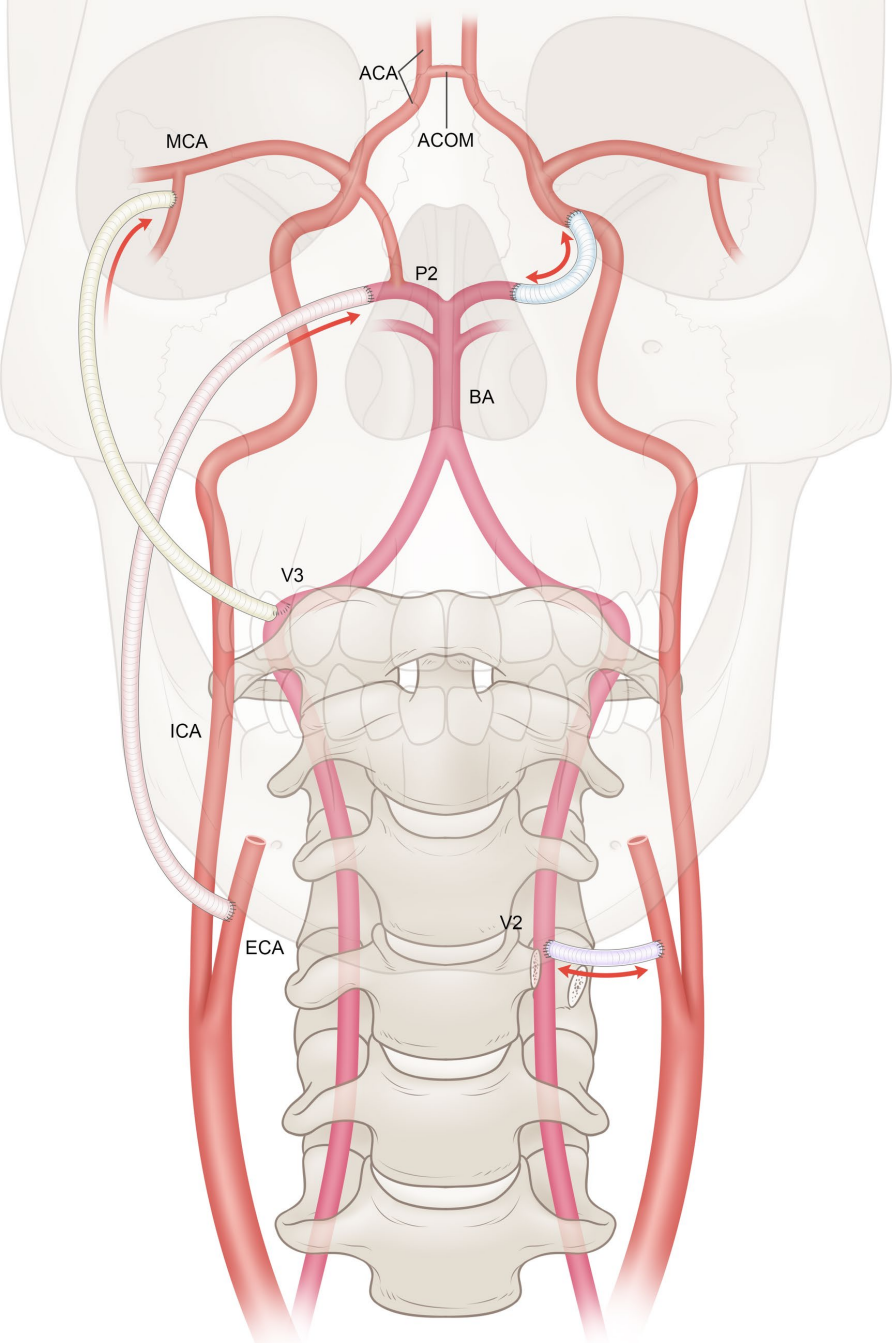
Schematic diagram of high-flow bypass techniques connecting the anterior and posterior circulations. Long-graft bypasses (left) include ECA-to-V2 (purple) and supraclinoid ICA-to-P2 (blue). Short-graft “artificial PCOM” techniques (right) include ECA-to-P2 (pink) and V3-to-MCA (green). Arrows indicate possible flow directions

The V2 segment bypass connects the cervical carotid artery to the extracranial VA, creating a short, direct communication between anterior and posterior circulations that functions as an extracranial posterior communicating artery (PCOM) [36]. Some authors have achieved anatomical PCOM reconstruction using a short interposition graft from the supraclinoid internal carotid artery (ICA) or M2 segment to revascularize the basilar artery (BA) in cases of dolichoectatic basilar trunk aneurysms [21, 24], representing exemplary intracranial-intracranial (IC-IC) bypasses (Fig. 1, Table 1). In contrast, the extracranial-extracranial (EC-EC) nature of the V2 bypass confers several advantages: (1) the proximal donor vessel delivers higher-pressure flow [5], (2) it provides physiological orthograde inflow to the downstream vasculature, avoiding flow reversal uncertainties, and (3) it eliminates the need for skull base surgery, craniotomy, or intradural manipulation, utilizing a shallower surgical field without deep anastomosis [20].

**Table 1:** Characteristics of the available high-flow bypass modality connecting anterior and posterior circulation

Bypass modality	Donor site	Recipient site	Artificial PCOM	Bi-direction between anterior and posterior circulation	Graft length	Flow direction in posterior circulation	Bypass type
ECA-V2	Cervical carotid	V2	extradural PCOM	yes	short	orthograde	EC-EC
Supraclinoid ICA-P2	Supraclioid ICA or M2	P2	intradural PCOM	possible	short	retrograde	IC-IC
VA-MCA	V3	M2	no	no (P → A)	long	—	EC-IC
ECA-P2	ECA or double STA	P2	no	no (A → P)	long	retrograde	EC-IC

Although Schneider et al. referred to the V3–middle cerebral artery (MCA) bypass as a "PCOM bypass," [31] its long graft resembles traditional external carotid artery (ECA)–posterior cerebral artery (PCA) bypass [22, 32] without the benefits of low vascular resistance or ease of harvest. Importantly, unlike the bidirectional flow modulation in PCOM or V2 bypass, the V3–MCA bypass unidirectionally shunts flow from posterior to anterior, while the ECA–PCA bypass does the opposite (Fig. 1, Table 1).

This study investigates the V2 bypass as a communicating pathway, expanding its indications to include posterior circulation ischemia, subclavian steal syndrome, and compensatory terminal BA aneurysms.

## Methods

### Inclusion criteria and perioperative assessment

**Fig. 2:**
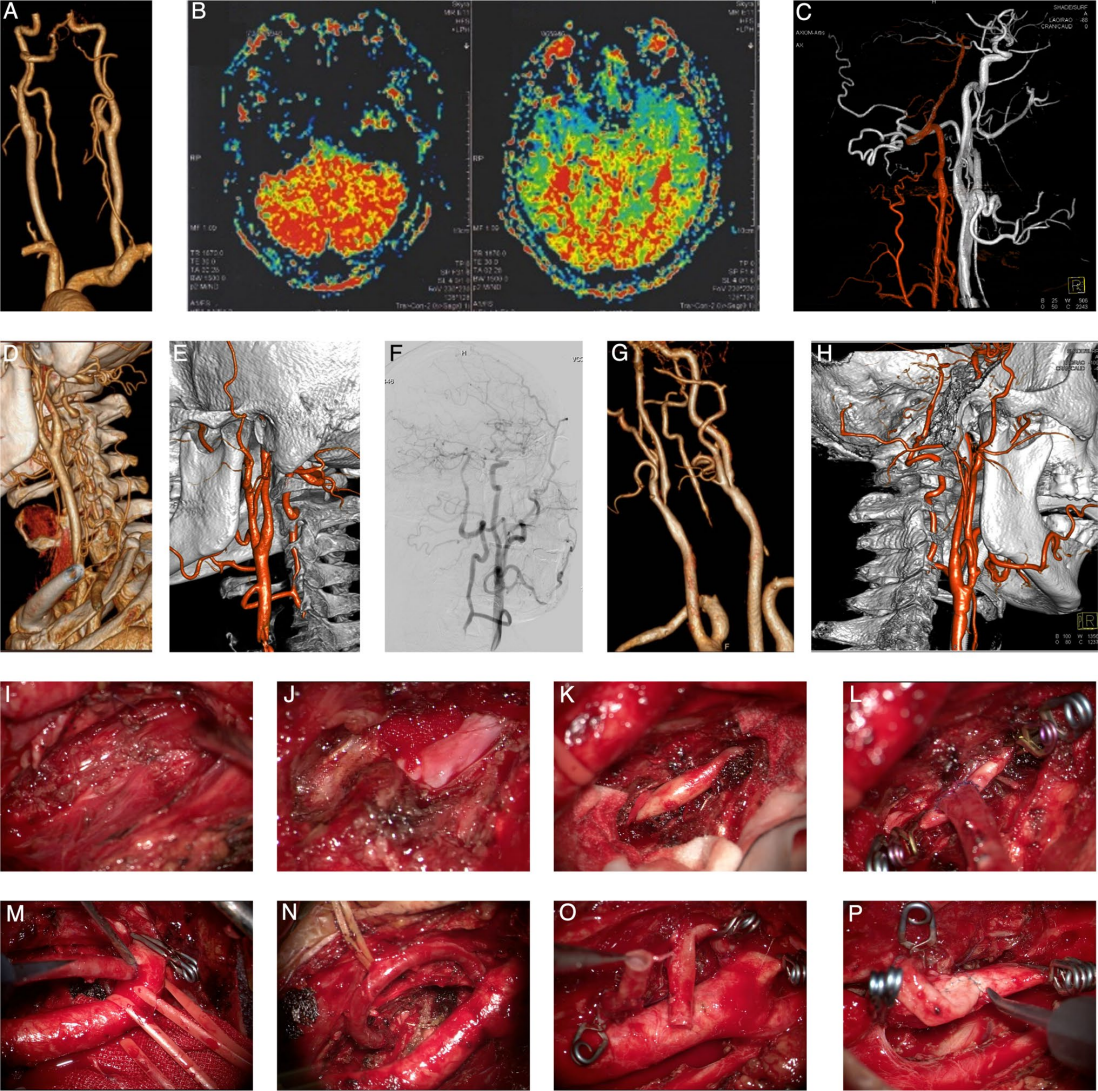
Representative cases of bilateral VA occlusions (Type I). A–F (Case 6): **A:** CTA reveals bilateral VA occlusions (left extracranial, right intracranial). **B:** MR perfusion indicates significant posterior circulation hypoperfusion. **C:** Preoperative angiography shows distal VA filling via OA and AA/DA collateral pathways. **D:** CTA demonstrates surgical anatomy of the cervical region. **E:** After initial occlusion, the donor site was switched from ECA to CCA, with subsequent angiography confirming patency. **F:** Postoperative angiography shows enhanced and timely filling of the distal vasculature. G–H (Case 7): **G:** CTA displays bilateral VA occlusions and severe ipsilateral ICA origin stenosis. **H:** Combined CEA and V2 bypass were performed utilizing overlapping surgical exposures. I–P (Intraoperative Sequence): **I:** The prevertebral aponeurosis is opened and retracted anteriorly. **J:** The intertransverse segment of V2 is identified deep to the muscle layer. **K:** The VA is exposed after unroofing the transverse foramen; hemostasis is aided with gelatin sponge or Surgicel. **L:** End-to-side anastomosis between the radial graft and V2 is completed. **M:** The graft is transposed to the pre-carotid space and anastomosed to the ECA. **N:** Final view after bypass completion. **O:** The occluded graft is examined and flushed after unsuccessful intraoperative angiography. **P:** Graft reimplantation to the CCA is performed as a salvage procedure

**Fig. 3:**
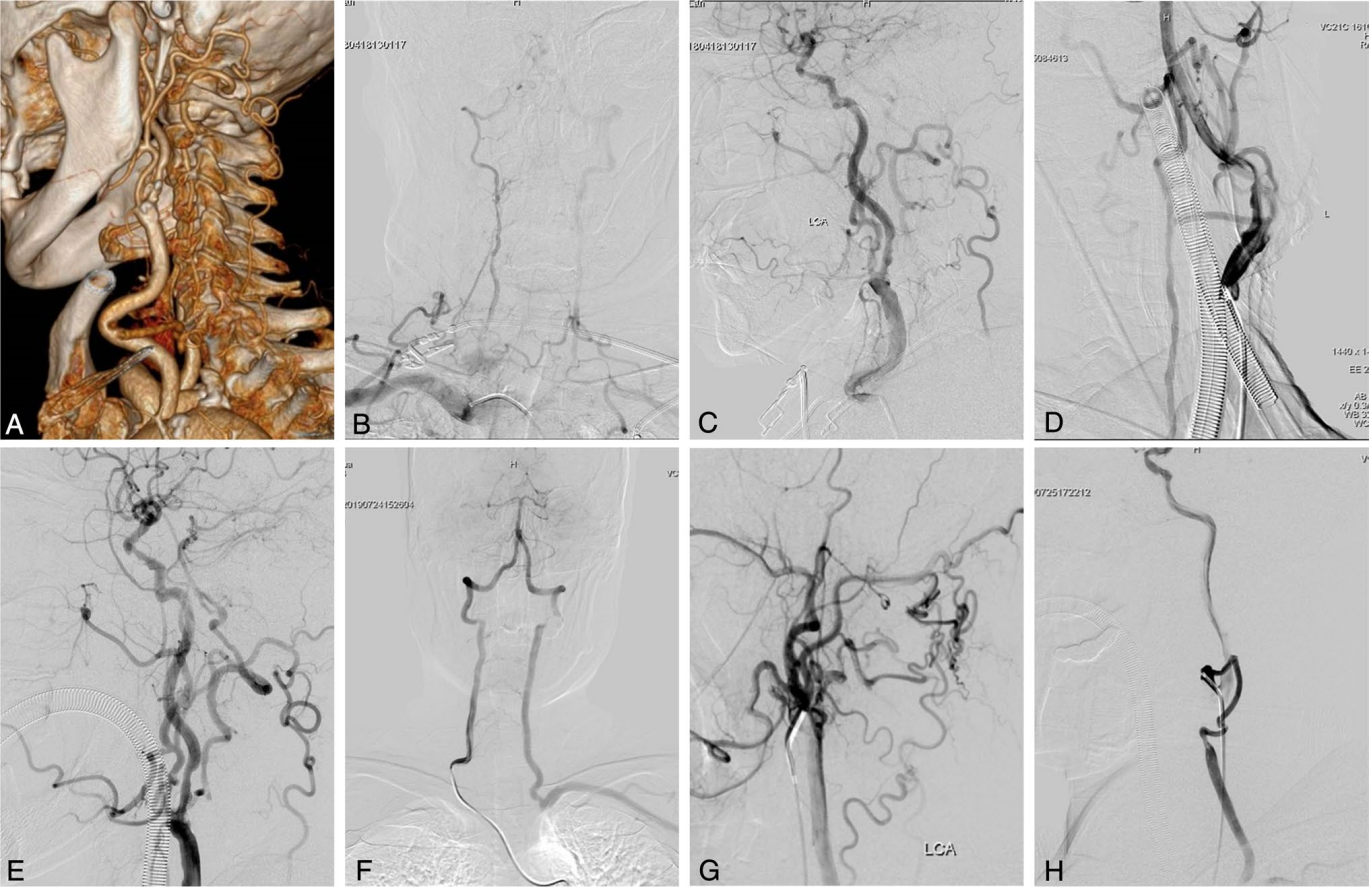
Cases of subclavian steal due to SBA occlusion (Type II). A–E (Case 2): **A:** CTA shows the occluded left SBA with a residual stump. **B:** Angiography reveals a hypoplastic contralateral VA with intracranial occlusion and minor steal via intervertebral branches. **C:** The OA serves as a collateral pathway, though flow is largely diverted through VA and AA/DA steal routes. **D:** Initial bypass flow is compromised due to persistent steal. **E:** After VA ligation, steal is markedly reduced, with posterior circulation supply restored. **F**–**H** (Case 5): **F:** Preoperative angiography demonstrates a well-developed right VA contributing pronounced steal; endovascular recanalization failed. **G:** OA collaterals provide minimal filling to the vertebrobasilar system. **H:** Without VA ligation, bypass flow participates in the steal phenomenon

**Fig. 4:**
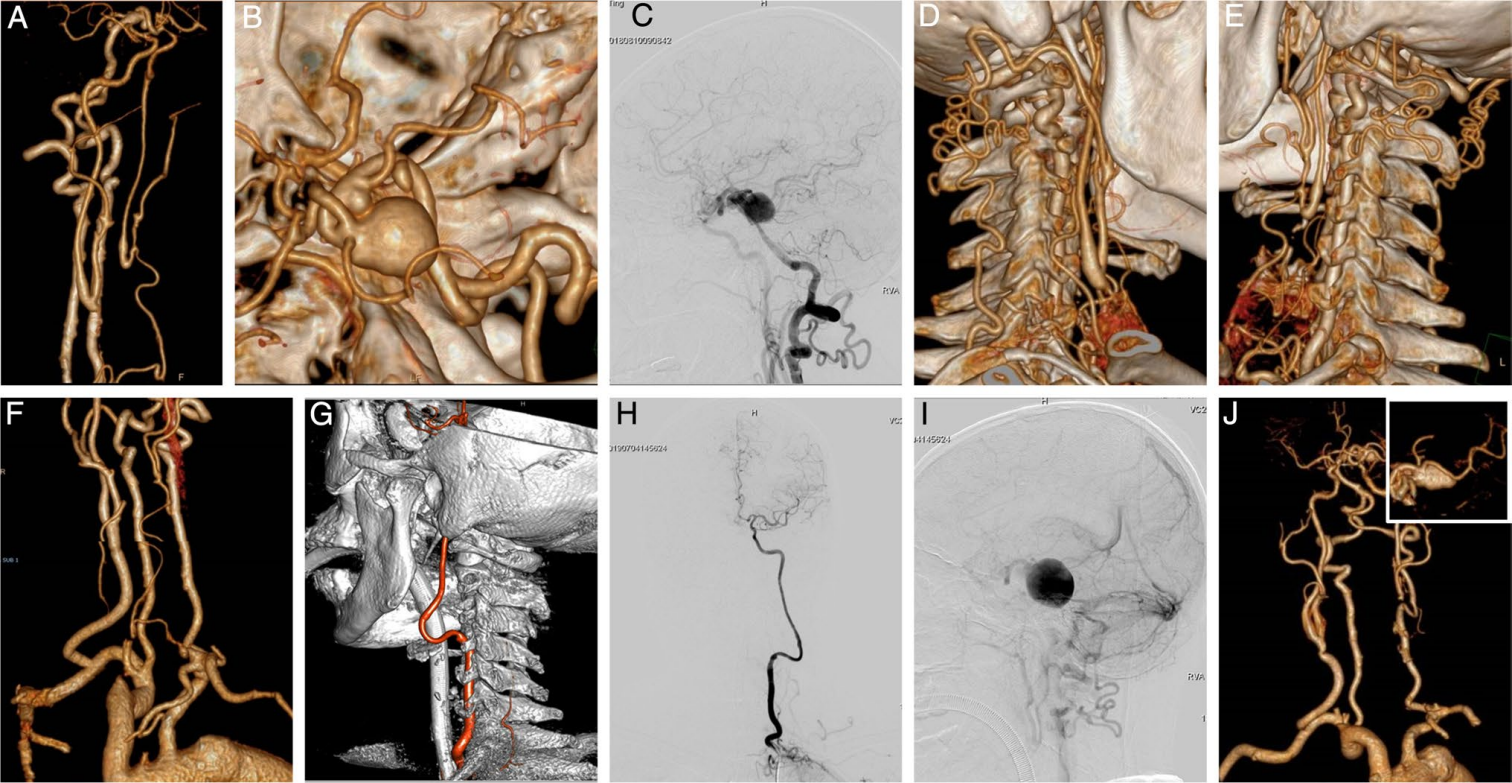
A case of compensatory terminal BA aneurysm (Type III). **A–B:** CTA confirms bilateral CCA occlusions and a giant terminal BA aneurysm involving the left PCA. **C:** Angiography shows flow diversion via the left PCOM and ACOM, contributing to aneurysm development. **D–E:** CTA reveals opened cervical collateral pathways via OA, AA/DA, and SBA branches. **F:** One-year patency of the venous graft bypass for right CCA reconstruction. **G–H:** Intraoperative angiography confirms graft patency with robust filling of the left ICA territory. **I:** Contrast retention is observed within the aneurysm sac. **J:** Follow-up CTA demonstrates progressive aneurysm shrinkage

This study was approved by the local ethics committee, and all patients provided informed consent. Preoperative assessment included the National Institutes of Health Stroke Scale (NIHSS) by an independent neurologist. Surgical indications were: Type I: Symptomatic bilateral VA occlusions (Fig. 2A–H); Type II: SBA occlusion with steal phenomenon and a well-developed ipsilateral VA (Fig. 3A–H); Type III: Compensatory terminal BA aneurysm due to bilateral common carotid artery (CCA) occlusions (Fig. 4A–J).

Inclusion criteria for posterior circulation ischemia were: Recurrent ischemic events in the vertebrobasilar territory refractory to medical therapy; Bilateral VA occlusion; CTA demonstrating vertebrobasilar visualization distal to steno-occlusion; DWI-confirmed infarction in the vertebrobasilar territory; MRP evidence of hypoperfusion correlating with angiographic findings; mRS ≤ 3; Age 40–80 years; Absence of severe cardiac disease.

MRI was used to identify cerebral ischemic lesions. DSA and CTA assessed vascular anatomy and hemodynamics. Aneurysmal changes were monitored in Type III cases. MR perfusion evaluated cerebral perfusion status. Follow-up began at 6 months postoperatively and annually thereafter, including NIHSS assessment and DSA/CTA for graft patency.

### Surgical techniques

All procedures were performed in a hybrid operating room. Patients were positioned supine with the head turned contralaterally and slightly extended. Excessive rotation was avoided to prevent vertebral body obstruction of the V2 segment. Dissection proceeded along the medial SCM to open the carotid sheath, exposing the carotid artery and internal jugular vein (IJV). The plane between these vessels was developed to access the retro-carotid space. The prevertebral aponeurosis was opened longitudinally to expose the longus colli muscle. The sympathetic trunk was carefully preserved.

The intertransverse portion of V2 between C3/4 or C4/5 was identified and used as a landmark to safely unroof the transverse foramen and expose the intraforaminal V2 segment [2, 3, 10]. The VA venous plexus was controlled with bipolar coagulation, gelatin sponge, or Surgicel. The radial artery was harvested and anastomosed end-to-side to the VA and ECA. In select cases, end-to-end anastomosis to the transected ECA was used to improve flow and patency. Graft patency was confirmed with indocyanine green videoangiography and intraoperative angiography (Fig. 2I–P, Video).

## Results

### Patient characteristics

A total of eight patients (6 males and 2 females) with a mean age of 62.4 years (range: 54–73 years) were included in this study. According to the classification established in our protocol, four patients (50%) presented with Type I pathology, three (37.5%) with Type II, and one (12.5%) with Type III. Detailed baseline and clinical characteristics are summarized in Table 2.

**Table 2:** Clinical features of our cases underwent V2 bypass

Classification	Case No	Sex/age	Lesion	Compensated collaterals	Management	Blood direction	Donor vessel	Recipient site of VA	Preoperative neurological symptoms	Imfarct extent
Type I	Case 1	M, 73	bilateral VA occlusions	OA, AA/DA	bypass	AC → PC	ECA	C4/5	visual blur, visual field defect	cerebellar hemisphere (r), fronto-parento-occipital lobe (r)
Type I	Case 6	M, 54	bilateral VA occlusions	OA, AA/DA, PCOM	bypass	AC → PC	CCA	C4/5	left limb weakness	cerebellar hemisphere (bi), occipital lobe(bi)
Type I	Case 7	M, 60	bilateral VA occlusions	AA/DA	bypass + CEA	AC → PC	ECA	C3/4	vertigo, dysdipsia, diplopia	cerebellar hemisphere (bi), occipital lobe(r)
Type I	Case 8	M, 54	bilateral VA occlusions	AA/DA	bypass + CEA	AC → PC	ECA	C3/4	vertigo, gait disturbance, bilateral lower limbs numb and weakness	medulla oblongata (r), cerebellar hemisphere (r), temporo-occipital lobe(l)
Type II	Case 2	F, 68	steal syndrome	OA	bypass + proximal occlusion	AC → PC	ECA	C3/4	vertigo, left limb numb and weakness	cerebellar hemisphere (bi), occipital lobe(bi), parental lobe (l)
Type II	Case 3	M, 58	steal syndrome	OA	bypass + proximal occlusion	AC → PC	ECA	C3/4	vertigo, right limb numb and weakness	pons, midbrain, temporo-occipital lobe (bi)
Type II	Case 5	M, 63	steal syndrome	—	bypass + proximal occlusion	AC → PC	ECA	C3/4	transient loss of consciousness	none
Type III	Case 4	F, 69	compensated BA aneurysm	PCOM, OA	right: SBA-SVG-CCA, left: bypass + distal occlusion	PC → AC	VA	C3/4	dizziness, headache	fronto-temporo-parental lobe (r)

### Preoperative cerebral hemodynamics

#### Type I

Collateral circulation was primarily maintained through pathways including the PCOM, occipital artery (OA) originating from the ECA, and ascending cervical artery/deep cervical artery (AA/DA) arising from the SBA. In certain cases, sequential filling via intervertebral branches between bilateral vertebral arteries was also observed (Table 2, Fig. 2A–C).

#### Type II

All three patients exhibited a well-developed vertebral artery on the side of SBA occlusion. Among them, two patients (Cases 2 and 3) had a non-dominant contralateral VA with associated stage I and II steal phenomena, respectively. Their hypoplastic contralateral VA was occluded intracranially and contributed negligibly to posterior circulation via indirect intervertebral steal. The OA served as the primary steal source through anastomoses with the ipsilateral VA and AA/DA (Fig. 3A–C). The remaining patient (Case 5) demonstrated bilateral symmetrical VAs with stage III steal, wherein the dominant contralateral VA constituted the major steal pathway, with minor participation from the ipsilateral OA, which did not substantively contribute to posterior circulation supply (Table 2, Fig. 3F–G).

#### Type III

In this singular case, preoperative CTA confirmed bilateral CCA occlusions and a giant aneurysm involving the terminal BA and left PCA (Fig. 4A–B). Compensatory supply to the cerebral hemispheres occurred via: (1) VA flow through the left PCOM and anterior communicating artery (ACOM) (Fig. 4C); (2) reversal of flow through OA anastomoses with AA/DA or VA, reconstituting ICA flow via the carotid bifurcation; and (3) small branches originating from the SBA directly connecting to the ECA (Table 2, Fig. 4D–E).

### Surgical procedures

Bypass grafting with anterior-to-posterior flow direction was performed in Type I and II cases. In Type II, VA ligation proximal to the anastomosis was carried out to eliminate steal phenomena. For the Type III case, parent artery occlusion distal to the anastomosis was implemented to redirect flow toward the anterior circulation. The recipient VA segment was selected at the C3/4 level in six cases and at C4/5 in two cases (Fig. 2D–E). Procedures were performed on the dominant VA side in Type I, the affected side in Type II, and on the left (considering the thoracic duct anatomy) in Type III. Two Type I patients (Cases 7 and 8) with concurrent severe stenosis at the origin of the ipsilateral ICA or mid-CCA underwent combined carotid endarterectomy (CEA) and V2 bypass surgery (Fig. 2G–H).

### Outcomes

1. Intraoperative angiography confirmed patent anastomoses in all cases except one (Case 6), in which the donor site was successfully switched from the ECA to the CCA, achieving final patency after re-anastomosis (Fig. 2E, O–P).
2. In Type I cases, DSA demonstrated adequate blood supply to the entire posterior circulation in all four patients (Fig. 2F). Among Type II cases, Cases 2 and 3 exhibited improved opacification of the intracranial vertebrobasilar system following anastomosis. However, persistent steal pathways initially diverted bypass flow (Fig. 3D). Subsequent VA ligation eliminated significant steal, effectively restoring posterior circulation perfusion with only minimal residual steal through AA/DA collaterals (Fig. 3E). In Case 5, without VA ligation due to personal reasons, bypass flow continued to participate in the steal phenomenon, merging with retrograde flow from the contralateral VA toward the SBA (Fig. 3H).
3. Except for Case 5, all patients with posterior circulation ischemia (Types I and II) showed symptomatic improvement and enhanced cerebral perfusion on postoperative imaging (Fig. 2B), with no recurrent ischemic events during follow-up. The mean NIHSS score improved significantly from 3.4 ± 1.0 preoperatively to 1.6 ± 0.7 postoperatively. Long-term graft patency was confirmed in all cases. Six patients achieved complete symptom resolution (mRS 0–1), while two exhibited functional improvement despite mild residual disability (mRS 2). In the Type III patient, follow-up CTA after one year demonstrated a patent SBA–CCA graft (Fig. 4F). Following left V2 bypass and VA ligation, the patient remained in good clinical condition (Fig. 4G–H), with intraoperative angiography revealing contrast retention within the aneurysm (Fig. 4I). Subsequent imaging confirmed progressive shrinkage of the aneurysmal sac (Fig. 4J).

## Discussion

### Surgical indications

#### Bilateral VA occlusions

Current guidelines for bypass surgery largely derive from anterior circulation ischemia, recommending quantitative perfusion parameters within the MCA territory. However, the vertebrobasilar system exhibits a distinct hemodynamic architecture. We propose a topographic division of the posterior circulation into upper (occipitoparietal regions and midbrain) and lower (cerebellar hemispheres and pontomedullary areas) territories. Bypasses such as the superficial temporal artery (STA)–superior cerebellar artery/PCA primarily augment perfusion at the basilar apex, particularly the posterior thalamoperforating arteries. Hypoperfusion in the upper territory may thus serve as an indicator for intervention. Conversely, OA–PICA bypass aims to improve perfusion in the lower territory (PICA and VA perforators) while enhancing overall posterior circulation supply [13]. Accordingly, we advocate patient selection based on demonstrated infarcts and hypoperfusion affecting both territories (Table 1).

Traditional techniques remain limited by patient comorbidities, deep anastomosis challenges, and infection risks. Our team previously developed the OA–V3 EC–EC bypass, which offers a less invasive alternative without craniotomy [35]. However, the OA–V3 technique necessitates stringent hemodynamic criteria, caliber matching between donor and recipient, and sometimes complex anastomotic configurations (e.g., double-barrel bypass) to ensure patency [35].

Although the V2 bypass requires an interposition graft and dual anastomoses, its high-flow capacity better meets the metabolic demands of the entire posterior circulation [4]. All four Type I cases in our series exhibited complete and orthograde filling of the posterior circulation. Exposure of the V2 segment—situated deeper than the carotid sheath—is straightforward, requiring only resection of the longus capitis muscle and unroofing of the transverse foramen. This facilitates combination with CEA (Fig. 2G–H) to address concurrent anterior and posterior circulation ischemia [11]. Moreover, compared with the OA–V3 bypass—which entails harvesting the OA through suboccipital musculature and navigating the suboccipital venous plexus—the V2 bypass offers a favorable alternative for surgeons less familiar with posterior cranial anatomy [35]. Currently, our center reserves the OA–V3 bypass for cases with inadequate recipient VA length distal to the occlusion [8, 11, 11, 22, 36].

#### The SBA occlusion with steal phenomenon

Endovascular recanalization for SBA occlusion carries risks including failure to cross sclerotic lesions (Fig. 2F), thromboembolism, vessel rupture, and dissection [1, 26, 29]. Hemodynamically significant steal can reconfigure native collaterals into steal pathways (Fig. 2C, G), necessitating effective intervention [30].

Selection for bypass requires a well-developed ipsilateral VA to sustain cerebral perfusion. Bypass flow is initially susceptible to steal; ligation of the VA proximal to the anastomosis is essential to abolish retrograde diversion (Fig. 2D–E). The magnitude of steal depends on contralateral VA dominance: a well-developed contralateral VA generates pronounced steal (Fig. 2F). In cases with robust steal, hemodynamic equilibrium may favor retrograde flow, diminishing the efficacy of the bypass (Fig. 2G-2H). In such scenarios, Hunterian ligation of the ipsilateral VA with reliance on contralateral VA supply may be preferable. Our institutional criteria for V2 bypass in Type II patients therefore include: (1) a well-developed ipsilateral VA to ensure sufficient bypass flow and facilitate ligation; and (2) a non-dominant contralateral VA to limit steal impetus.

#### Compensatory aneurysms of upper posterior circulation

While the V2 bypass has been historically applied to cervical VA aneurysms [11], we pioneered its use in a terminal BA aneurysm. Unlike typical aneurysms associated with PCOM hypoplasia, this case exhibited a well-developed PCOM secondary to bilateral CCA occlusions, contributing to aneurysm formation. The V2 bypass served a dual role: intentional VA obstruction induced “flow reduction” within the aneurysm, while the graft diverted flow to revascularize the anterior circulation, thereby mitigating hemodynamic stress across the circle of Willis. This innovative application reorients conventional V2 bypass flow to posterior-to-anterior direction, transforming the graft into a dynamic modulator of trans-circulatory flow rather than a passive conduit [6].

This approach is particularly valuable in long-segment CCA lesions [25, 37], where the absence of suitable ipsilateral high-flow donors poses a challenge [17, 19]. Existing options such as the “Bonnet” bypass—which uses contralateral STA or ECA donors and requires a long graft traversing the calvarium to the MCA [7, 16, 28]—are hampered by extended graft length and vulnerability to mechanical damage [9, 23, 33]. The V3 bypass, acting as a “jump graft” between the V3 segment and intracranial recipients in far-lateral exposure, is more suitable when cervical carotid inflow is compromised [12, 27]. In our Type III case, the right side (avoiding the thoracic duct and sympathetic chain) and a low-lying CCA bifurcation permitted a shorter graft; we therefore employed a conventional SBA–CCA interposition bypass, which shares the surgical field with V1 segment exposure [14, 15, 18].

### Surgical considerations

#### Recipient site selection

The anterolateral cervical approach provides comprehensive exposure of the extradural VA through the anatomical plane between the SCM and IJV, ensuring direct visualization and safe access to the VA [10]. In our series, the mid-portion of the V2 segment (between C3 and C5) was preferentially selected as the recipient site due to its relatively straightforward anatomical relationships and absence of critical neurovascular structures [2, 3, 10].

While some authors advocate for using the V2 segment between C1–C2 as the recipient site [2, 4–6]—citing improved working angles and reduced depth of the surgical corridor—this approach presents notable challenges. The distal extracranial VA forms complex loops around the atlas and axis to accommodate cervical rotation, rendering this segment more superficial and providing over 2.0 cm of mobility without requiring foramen unroofing [6]. However, surgical exposure at this level demands meticulous dissection of high cervical musculature and carries increased technical complexity. Extreme contralateral head rotation, though improving exposure, elevates the risk of compressing the ICA and IJV, [4] occasionally necessitating jaw subluxation to maintain a neutral head position [6]. Furthermore, this approach introduces longer graft requirements and involves intricate regional anatomy without conferring substantial advantages over the well-established OA-extracranial VA bypass.

The mid-V2 segment offers distinct advantages over both superior (C1–C2 and V3) and inferior (V1) segments. Its isolation from vital structures minimizes risks associated with superior exposures—such as injury to the accessory nerve—and avoids complications related to inferior exposures, including damage to the vagus nerve, stellate ganglion, recurrent laryngeal nerve, as well as potential chylous fistula or pneumothorax. Intraoperatively, we routinely incise the prevertebral aponeurosis laterally and reflect it anteriorly to preserve the integrity of the sympathetic trunk. Principal technical challenges during V2 exposure include: (1) controlling hemorrhage from the extensive venous plexus, which requires careful management using gelatin sponge compression and precise bipolar coagulation, and (2) identifying and preserving vertebral artery loops—a common anatomical variant—during dissection of the intertransverse segment to prevent iatrogenic injury.

#### Donor site considerations

The optimal donor site selection remains subject to ongoing debate, reflecting competing hemodynamic and technical priorities. Carney et al. emphasized the influence of head positioning on graft geometry, recommending alignment parallel to the neck axis to minimize tension [5]. This approach favors using the CCA as the donor when anastomosing to high cervical V2 segments (C1–C2), as detailed in Table 3. Conversely, Kakino et al. argued that elongated grafts increase susceptibility to twisting and kinking, advocating instead for shorter grafts between the ECA and mid-cervical V2 (C3–C5) to mitigate the effects of cervical motion (Table 3) [20].

**Table 3:** Different reconstructed configurations of V2 bypass

Authors	Bypass modality	Bypass configuration
Present study	ECA-RA-C3/C5 V2	horizontal
Carney [5], Camp [4], Chwajol [6]	CCA-SVG-C1/C2 V2	parallel
Kakino [20]	ECA-SVG-C3/C5 V2	horizontal
Gerke [11]	ECA-C3/C5 V2	transposition
Diaz [20]	CCA-SVG-C3/C5 V2	diagonal

Diaz offered a third perspective, supporting CCA donation for mid-V2 bypass to maintain antegrade flow orientation and avoid turbulent flow patterns associated with acute graft angles (Table 3) [20]. Our institutional experience incorporates elements of all three approaches: when intraoperative patency appears compromised, we employ a salvage maneuver involving proximal transection of the radial artery graft and reimplantation from the ECA to the CCA to reestablish antegrade perfusion (Fig. 1E–F). Additionally, we recognize that the steep-angled configuration of CCA-distal V2 bypass grafts may be particularly suitable for hybrid operating settings, providing an optimal trajectory for potential endovascular interventions [6].

## Conclusion

The cervical carotid-to-V2 vertebral artery bypass effectively establishes a high-flow, extracranial communication between circulations, avoiding complex skull base surgery. It successfully addresses vertebrobasilar ischemia, subclavian steal, and select aneurysms by restoring perfusion and modulating flow. The technique demonstrates notable efficacy, procedural reproducibility, and holds promise for broader clinical adoption.

## Supplementary Information

Below is the link to the electronic supplementary material.

Supplementary file1 The video illustrates the surgical procedure of an extracranial posterior communicating bypass performed for bilateral VA occlusions (MP4 258724 KB)

## Data Availability

The data and materials supporting this study are available from the corresponding author upon reasonable request.

## Abbreviations

AA: Ascending cervical artery
ACOM: Anterior communicating artery
BA: Basilar artery
CCA: Common carotid artery
CEA: Carotid endarterectomy
DA: Deep cervical artery
ECA: External carotid artery
ICA: Internal carotid artery
IJV: Internal jugular vein
MCA: Middle cerebral artery
OA: Occipital artery
PCOM: Posterior communicating artery
PICA: Posteroinferior cerebellar artery
SBA: Subclavian artery
SCM: Sternocleidomastoid muscle
STA: Superficial temporal artery
VA: Vertebral artery
